# *VibeComm*: Radio-Free Wireless Communication for Smart Devices Using Vibration

**DOI:** 10.3390/s141121151

**Published:** 2014-11-10

**Authors:** Inhwan Hwang, Jungchan Cho, Songhwai Oh

**Affiliations:** Department of Electrical and Computer Engineering and ASRI, Seoul National University, 1 Gwanak-ro, Gwanak-gu, Seoul 151-744, Korea; E-Mails: inhwan.hwang@cpslab.snu.ac.kr (I.H.); jungchan.cho@cpslab.snu.ac.kr (J.C.)

**Keywords:** smartphones, vibration, wireless, communication

## Abstract

This paper proposes *VibeComm*, a novel communication method for smart devices using a built-in vibrator and accelerometer. The proposed approach is ideal for low-rate off-line communication, and its communication medium is an object on which smart devices are placed, such as tables and desks. When more than two smart devices are placed on an object and one device wants to transmit a message to the other devices, the transmitting device generates a sequence of vibrations. The vibrations are propagated through the object on which the devices are placed. The receiving devices analyze their accelerometer readings to decode incoming messages. The proposed method can be the alternative communication method when general types of radio communication methods are not available. *VibeComm* is implemented on Android smartphones, and a comprehensive set of experiments is conducted to show its feasibility.

## 1. Introduction

With the advent of the smartphone and the growth of smartphone users, short-range wireless communication between smartphones and between a smartphone and other devices has become an important capability of smartphones. A number of short-range wireless communication methods have been developed and embedded into smartphones, including Wi-Fi, Bluetooth and near-field communication (NFC). However, the currently available short-range radio communication methods cannot be operated when just one of the communication devices is not equipped with radio communication components. In this paper, we propose a new short-range wireless communication method, named *VibeComm*, which utilizes the built-in vibrator and accelerometer in smartphones. The proposed method is motivated by the old-fashioned dot-and-dash communication method or Morse code, and it can show the possibility of a noveltype of wireless communication method without using radio.

We assume that the communicating smart devices are placed on a single object, such as a table or a desk. The transmitting device generates a sequence of vibrations, which is similar to Morse code. The generated vibrations are propagated to the other devices through the object on which the devices are placed. The receiving devices decode the incoming message based on their accelerometer readings. The proposed method can be used to communicate with devices with accelerometers, and no radio is required. Hence, the proposed method enables communication with a wide range of everyday objects that lack radio functionality, closing the gap between the cyber world and the physical world.

Compared to the existing short-range wireless communication methods, such as near-field communication (NFC), radio-frequency identification (RFID), Wi-Fi, ZigBee (IEEE 802.15.4) and Bluetooth, the proposed method has a number of desirable characteristics. NFC is used for short-range communication with a maximum communication range of 20 cm based on RFID technology [1]. While a number of smartphone manufacturers are starting to package NFC chips in their smartphones, NFC is not yet widely available. In addition, NFC's practical working distance is about 4 cm, while 20 cm is the theoretical maximum limit. The proposed method *VibeComm* can provide a longer communication range than NFC. Furthermore, two-way and one-to-many communication are available in *VibeComm*. RFID is another wireless technology for non-contact communication using radio [2], but it suffers from similar shortcomings as NFC, as well as the cost of adding RFID tags is not negligible [3]. While Wi-Fi [4], Bluetooth [5] and ZigBee [6] are widely available for longer range communication than NFC, they still need extra components that make it possible to communicate [7]. Infrared data association (IrDA) was a widely-used short-range communication method for personal digital assistants (PDAs) and mobile phones before the introduction of smartphones. IrDA uses infrared to enable mobile phones to send and receive data up to 115 kb/s. However, its communication range is less than 1 m, and its angular coverage is between a 15- and 30-degree half-angle cone from the optical axis of the receiver device [8].

A number of non-radio-based communication methods have been proposed before. Komine *et al.* [9] proposed a new communication method based on LED lights. However, it requires a line of sight between communicating devices, and one-to-many communication is not possible without an extra apparatus. Arentz *et al.* [10] proposed the use of sound in the frequency range from 20 kHz to 23 kHz as an alternative to IrDA. However, the sound can be easily overheard, and the method is not robust against ambient noise. Chang *et al.* [11] introduced *ComTouch*, which allowed communication utilizing vibration. Their method was also motivated by Morse code, but they used the vibration signal to replace characters or sounds, especially for deaf/blind people. They presented a custom-made device that could generate vibrations. A sender sends a message by grabbing the device, and the received message is presented as a series of vibrations. Studer *et al.* [12] proposed *Shot* for secure communication. It requires direct contact between two devices, and they mutually communicate for identification using vibration. However, the actual message is transmitted using radios, unlike *VibeComm. VibeComm* can be easily modified to transmit predefined vibration patterns to help communication between a smartphone and a deaf/blind person.

With various sensors in a smartphone, such as an accelerometer, digital compass, gyroscope, GPS, microphone and camera, a number of researchers have applied various sensing data collected by smartphones to discover contexts about a user and her surroundings to deliver better services to the users. For example, a vibrator and an accelerometer in a smartphone, a combination that is utilized in *VibeComm* for communication, has been applied to the indoor localization problem [13,14]. Shafer [13] used a vibrator and accelerometer to classify seven pre-defined locations using a support vector machine with time-series accelerometer readings. Kunze *et al.* [14] proposed a symbolic localization method through active sampling of acceleration and sound signatures. Their method used vibration and sound (short and high frequency beeps) to sample the response of the environment.

As an alternative to radio-based wireless communication, Yonezawa *et al.* [15] proposed a method to transfer data from a smartphone to a notebook by generating vibrations from a smartphone. It is assumed that a smartphone is directly in contact with a notebook for communication [15]. In [16], the authors applied the method from [15] for pairing electronic devices. Again, two communicating devices are required to be directly in contact with each other. *VibeComm* is similar to [15], since it uses a vibrator and an accelerometer, but *VibeComm* does not require direct contact between devices, as well as two-way or one-to-many communication is possible in *VibeComm*. Since the vibration energy is dissipated in the object on which communicating devices are placed and there are echoes from object boundaries as vibrations bounce back, a more sophisticated communication system design is required for *VibeComm*.

The proposed method is ideal for low-rate off-line communication. As its communication medium is the object on which devices are placed, communication over a longer distance and broadcasting to multiple devices are possible using *VibeComm*. These features distinguish the proposed method from [15,16] and radio-based communication methods. One possible application of *VibeComm* is the task of configuring a large number of devices simultaneously without using radios. For instance, consider a desk that is used by multiple users. Suppose that a number of electronics, such as a notebook, a digital alarm clock, a tablet, an MP3 player, a telephone and a TV, are placed on the desk, and each electronic device on the desk has predefined user settings. If each user has her preferred settings for all electronics stored on her smartphone, then the proposed method can be used to configure all electronics on the desk for each user by simply placing the user's smartphone on the desk.

The remainder of this paper is organized as follows. An overview of the proposed method is shown in Section 2. Section 3 discusses issues when designing the proposed method, and Section 4 describes the design of *VibeComm*. The implementation of the proposed method is described in Section 5, and the results from the experiments are discussed in Section 6. Two-way and one-to-many communication demonstrations are given in Section 7, and remaining issues are discussed in Section 8.

## 2. *VibeComm* Overview

**Figure 1.**
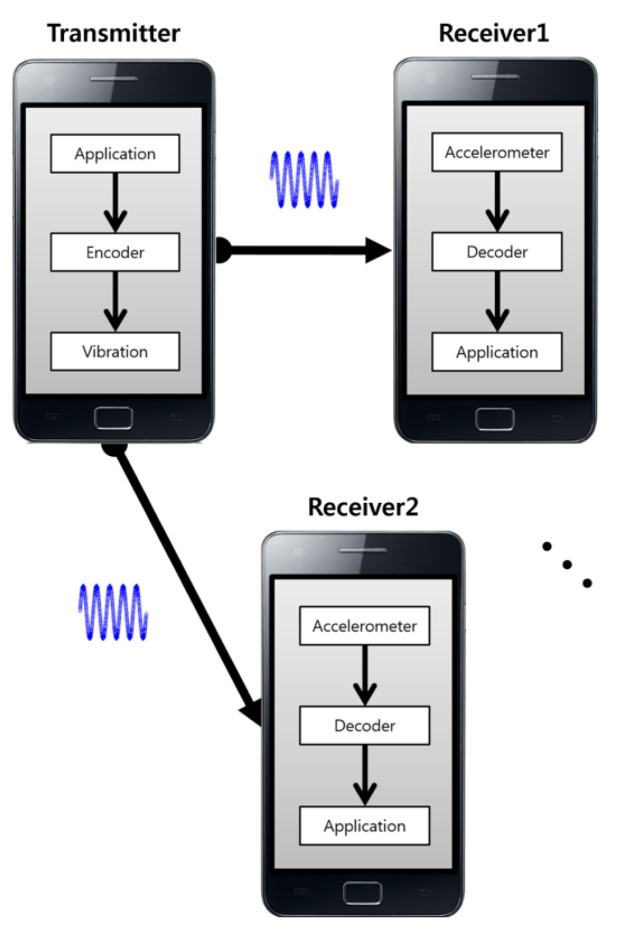
An overview of *VibeComm*. A transmitter encodes a message into a series of vibrations. Vibrations propagate through the object on which the transmitter is placed. Receivers that are placed on the same object listen for incoming vibrations using accelerometers and decode them into messages.

An overview of *VibeComm* is shown in Figure 1. We consider the problem of packet-based communication between smart devices that are equipped with accelerometers and vibrators. The transmitting device takes an input from a user application and encodes the message into a series of packets. Each packet consists of frames, and each frame represents a bit of information. Each packet is transmitted from a transmitter by generating vibrations. A receiving device listens for incoming vibrations using its accelerometer and decodes incoming packets. The decoded message is then delivered to a user application. Due to the current hardware limitations discussed below, a sophisticated coding scheme cannot be applied. A simple coding scheme is applied to *VibeComm*, where the presence of vibration in a frame indicates a bit 1 and the absence of vibration represents a bit 0. In order to reduce power consumption, impulse-like vibration signals are used to encode a packet, instead of continuous vibrations. In this paper, we focus on sending and receiving text messages to demonstrate the feasibility of vibration-based wireless communication. In the next two sections, we discuss design issues, calibration of raw accelerometer readings and the design of the transmitter and receiver for *VibeComm*.

## 3. Design Considerations

We have encountered a number of issues while developing the proposed system, and they are time synchronization, inconsistent sampling frequencies, effects of echoes and detection sensitivity. *VibeComm* is designed to address these issues.

### 3.1. Time Synchronization and Inconsistent Sampling Frequency

Time synchronization between a sending device and a receiving device is critical for reliable communication. When time synchronization fails, a frame can be read incorrectly, and a bit error will result. Unfortunately, the wall clock available in smart devices is not sufficient, since there can be an unknown length of delay from the propagation of the vibration signal. Hence, it is required to synchronize time between a transmitter and receivers each time communication starts for successful communication.

In addition to time synchronization, we have observed that the sampling frequency of an accelerometer is inconsistent. Android-based smartphones support four different sampling rates for their accelerometers, and they are *SENSOR_DELAY_NORMAL*, *SENSOR_DELAY_UI*, *SENSOR_DELAY_GAME* and *SENSOR_DELAY_FASTEST*. We have empirically measured each sampling rate, and they approximately correspond to 6 ± 1 Hz, 17 ± 1 Hz, 50 ± 1 Hz and 100 ± 1 Hz, respectively. Furthermore, the sampling rate varies between models. We have also observed that both the sampling rate and the sampling interval vary depending on the load and state of the Android OS. Hence, it is not possible to sample with an exact sampling frequency with current Android platforms.

**Figure 2.**
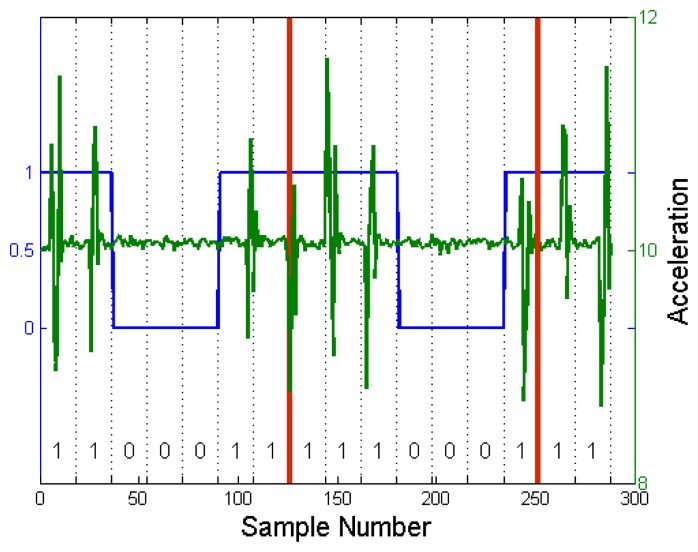
A successive transmission of 1100011. A bit error occurs due to time synchronization and an inconsistent sampling frequency.

An example is shown in Figure 2. We have transmitted a successive bit-stream of 1100011 from the sending device and examined the received data from the receiving device. Note that we have assumed that the sampling rate of the accelerometer is 17 Hz. The first seven bits are decoded correctly. However, the next seven bits are decoded as 1110001, which is a one-bit shifted version of the transmitted signal. This shows that a small misalignment can accumulate and cause a decoding error.

As can be seen from Figure 2, the received vibration signal is not located exactly in the middle of a frame, although it was transmitted in the middle of a frame. Actually, the location of the vibration signal drifts to the right as time passes. Since the direction and magnitude of the drift is not fixed, we have to regularly synchronize the clocks of the transmitter and receivers.

### 3.2. Effects of Echoes

As the vibration signal propagates throughout the communication medium object, the signal can be reflected from boundaries and other objects placed on the communication medium. These reflected vibration signals can make decoding more challenging. We can reduce the effect of echoes by generating vibration for a short period of time. An example can be seen from Figure 2. In addition, this impulse-like vibration signal can reduce the power consumption of the transmitter. While the effect of echoes was not considered in [15,16], it becomes a significant issue in *VibeComm*, as it is designed for communication over a longer distance through a rigid material.

### 3.3. Detection Sensitivity

**Figure 3.**
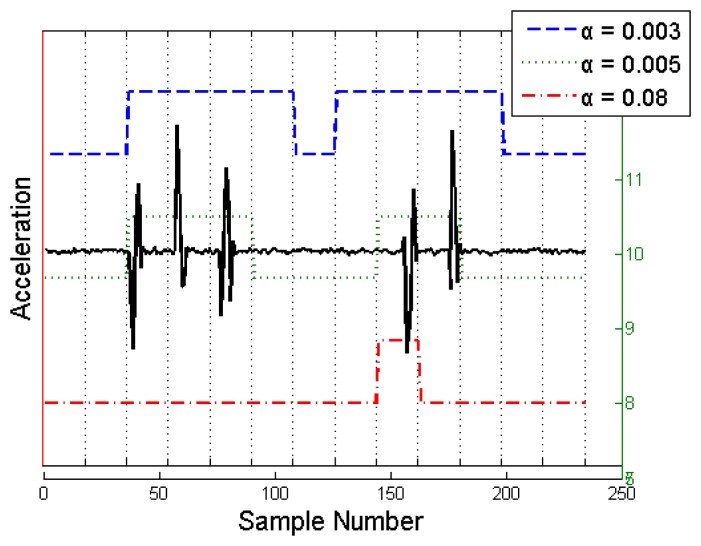
An effect of the threshold *α* from Equation (3) on decoding.

Since the vibration signal strength varies depending on the communication medium type (the rigidness of the medium) and decays as the communication distance increases, the signal detection sensitivity has to be set appropriately. Unexpected circumstantial factors, such as interfering vibration introduced by other objects, can make this problem even more difficult. The problem resulting from the rigidness of the communication medium can be solved to some degree by adjusting the detection threshold. As shown in Figure 3, the choice of the threshold value can make significant differences in decoded messages. The results shown in Figure 3 were obtained from a rigid wooden table, and the distance between the sending device and the receiving device was 50 cm. The threshold used in the figure is *α* from Equation (3). See Section 4.3 for more details about how a vibration signal is converted into a binary symbol. As shown in Figure 3, when the threshold gets smaller, the movement detection gets more sensitive. When the threshold gets larger, the movement detection is less sensitive and robust against disturbances. However, less sensitivity may cause the decoder to miss a signal from the transmitter. Hence, the threshold adjustment based on the communication medium type and ambient disturbances is important for the success of *VibeComm*.

### 4. *VibeComm* Design

In this section, the design of *VibeComm* is presented, including accelerometer calibration, the structure of a packet, the design of the transmitter and receiver, the design of a superframe, vibration signal shaping and adaptive threshold adjustment methods.

### 4.1. Accelerometer Calibration

In order to compensate for the hardware variation of an accelerometer in each smartphone, we need to convert raw accelerometer readings into the standard G unit using device-specific parameters. We use the normalization scheme proposed in [17], which is required only once for each smartphone.

A user is asked to hold his or her smartphone still and oriented towards different directions, which are not necessarily aligned with the direction of gravity. After a sufficient number of samples are collected, the normalization parameters are estimated as follows.

Let **a** = (*a_x_*, *a_y_*, *a_z_*) be a raw accelerometer reading and **n** = (*n_x_*, *n_y_*, *n_z_*) be the normalized accelerometer reading along each axis in unit G. The following function is used in normalization:

(1) $$f\left(n_{x},n_{y},n_{z}\right)=\sqrt{n_{x}^{2}+n_{y}^{2}+n_{z}^{2}}$$

(2) $$n_{\text{axis}}=K_{\text{axis}}a_{\text{axis}}+b_{\text{axis}}$$

where *K_x_*, *K_y_*, *K_z_* are the respective scaling factors and *b_x_*, *b_y_*, *b_z_* are the offsets of the accelerometer. When the phone is stationary, the function *f* is assumed to be one. Hence, to find the normalized accelerometer readings *n_x_*, *n_y_* and *n_z_*, we need to estimate parameters *K_x_*, *K_y_*, *K_z_*, *b_x_*, *b_y_* and *b_z_*, which make the function *f* unity when the phone is stationary. In order to solve this parameter estimation problem, we use a least squares estimator based on the linear approximation of function *f* [17]. To get these parameters, we collected 2000 accelerometer data in the ±*x*, ±*y* and ±*z* direction while the smartphone is not moving. By doing this, we can minimize the error occurring due to the variation of devices.

### 4.2. Transmitter Design

**Figure 4.**
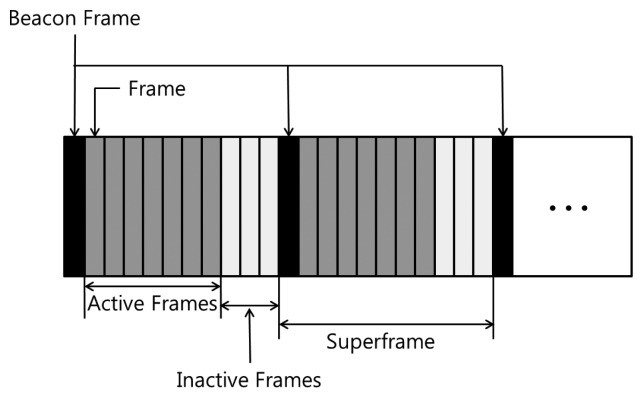
The structure of a superframe for *VibeComm*.

We consider packet-based communication in *VibeComm*, and a packet is contained in a superframe, a collection of frames. Each frame represents one bit of information. Figure 4 shows the structure of a superframe. In our current implementation, a superframe contains 11 frames.

Each superframe starts with a beacon frame, which is used to synchronize devices. A superframe consists of a beacon frame (one frame), active frames (seven frames) and inactive frames (three frames). The active frame field contains data, and seven frames are used in our current implementation to encode an ASCII character in each packet. The inactive frame field is a set of frames between the end of the active frame field and the start of the next superframe. While a lengthy inactive frame field is ideal in order to reduce the time synchronization error, we set the size of the inactive frames to be about a half of the size of the active frame field to improve the transmission rate. The effect of the inactive frame field will be discussed in detail in Section 4.4.

In our current implementation, each frame is one second long. The resulting transmission rate is about 0.64 bits per second (7 bits per 11 s) or 0.09 packets per second, and this is due to the limitation of the Android platform used in the experiments. Currently, stable vibrations are not possible on Android platforms, and we have tested if the duration of a vibration is shorter than 0.2 s. Therefore, considering the resting time to minimize the effect of echoes, the minimum size of each frame suitable for reliable communication is experimentally found to be one second. While the overall communication time is slow in our prototype, we believe that the problem will be addressed when more sophisticated and reliable vibrators are available in future smartphones. In order to transmit a bit 1, we generate an impulse-like vibration for 0.2 s at the center of a frame. No vibration in a frame means a transmission of 0. The inactive frames do not contain vibrations, and a beacon frame also transmits a 0.2 s-long impulse-like vibration. This impulse-like vibration signal is used to reduce the power consumption of the transmitter and to avoid the effect of echoes.

### 4.3. Receiver Design

The value *f* in Equation (1) represents the magnitude of a normalized accelerometer reading. In order to determine the existence of an incoming vibration signal, the magnitude of a normalized accelerometer reading is compared to the sample standard deviation. Let *B*(*i*) be the decoded binary value for the *i*-th frame. *B*(*i*) is determined as follows:

(3) $$B\left(i\right)=\{\begin{array}{cc} 1 & \text{if}\;\frac{\sigma_{i}}{\mu_{i}}\geq \alpha \\ 0 & \text{if}\;\frac{\sigma_{i}}{\mu_{i}}<\alpha \end{array}$$

where:

(4) $$\mu_{i}=\frac{1}{N}\sum_{j=1}^{N}n_{ij}$$

(5) $$\sigma_{i}=\sqrt{\frac{1}{N}\sum_{j=1}^{N}{\left(n_{ij}-\mu_{i}\right)}^{2}}$$

Here, *μ_i_* and *σ_i_* are the mean and standard deviation of *S_i_* = {*n_i_*_1_, *n_i_*_2_, …, *n_iN_*}, a set of normalized acceleration reading in the *i*-th frame, where *n_ij_* is the normalized acceleration reading of the *j*-th sample in the *i*-th frame, which is computed using Equation (1), and *N* is the number of acceleration readings in a frame. The parameter α is a threshold, which determines the detection sensitivity. Thus, the decoded binary value for the *i*-th frame is 1 if the ratio between the standard deviation and the mean of the frame is larger than the threshold, and otherwise, it is 0. A number of methods to adaptively adjust the threshold are proposed in this paper, and they are discussed in Section 4.6.

**Figure 5.**
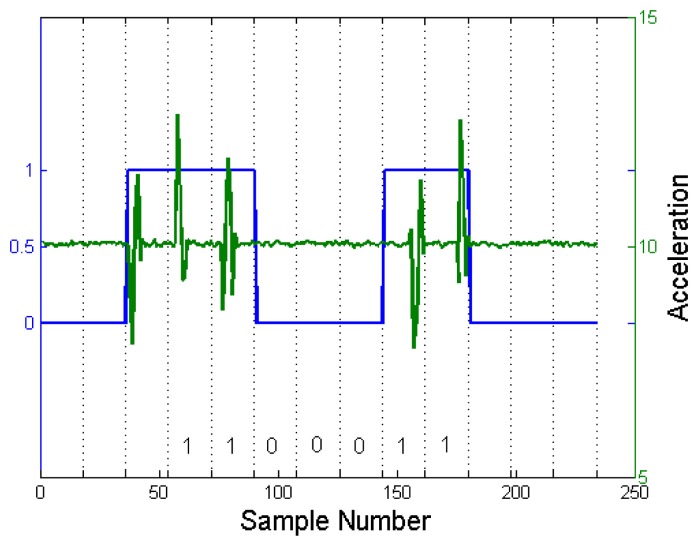
The *x*-axis indicates the successive input acceleration readings. The *y*-axis on the left side indicates the calculated binary signal for each frame, and the right side shows the raw accelerometer values.

Figure 5 shows an example with raw acceleration readings and detected binary bits for a single superframe. In Figure 5, each frame consists of 16 samples, and the detailed explanation for the frame size is discussed in Section 4.4.

### 4.4. Superframe Design

Since the accelerometer sampling rate and vibration intervals vary depending on the load and the state of the Android OS, it is impossible to synchronize the clocks of the transmitter and receivers. Since it is not trivial to synchronize each frame, we perform synchronization at the superframe level using a beacon frame and inactive frames. A beacon frame is used to notify receiving devices that there is an incoming packet and to synchronize the beginning of a packet. An example is shown in Figure 5. A packet is transmitted, including a leading beacon frame. The first frame is decoded as a beacon frame. The subsequent frames are decoded, and the receiver recovers 1100011. The use of the beacon frame also prevents error accumulation.

In addition to a beacon frame, an inactive frame field is padded to the end of the active frame field, as shown in Figure 4, to make the detection of a beacon frame easier. The detection of a beacon frame is easier with a longer inactive frame field, but a longer inactive frame field implies a longer transmission time. After a number of experiments, we have found that an inactive frame field of three frames is a good trade-off between speed and correctness when the active frame field contains seven frames.

As mentioned earlier, Android-based smartphones support four different settings for accelerometer sampling rates. We have decided to use *SENSOR_DELAY_UI*, which provides an approximate sampling rate of 17 ± 1 Hz, considering the energy efficiency and communication reliability This is the lowest sampling rate that can make it possible to operate *VibeComm* reliably While the average sampling rate is 17 Hz, the actual sampling rate changes over time depending on the load and state of the Android OS. We avoided this inaccuracy by adjusting our decoder to operate at 16 Hz, the slowest sampling rate for *SENSOR_DELAY_UI*.

### 4.5. Vibration Signal Shaping

**Figure 6.**
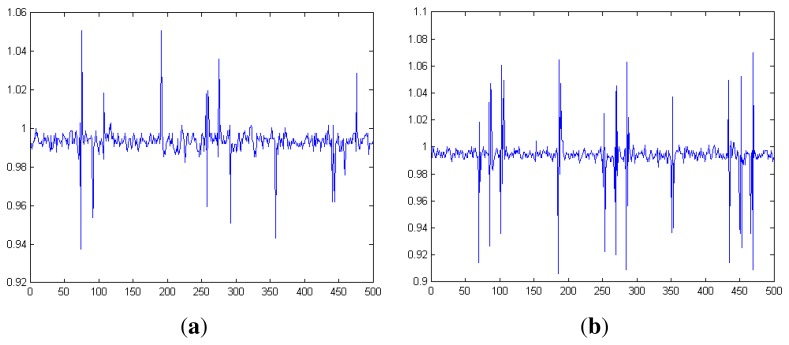
Normalized acceleration readings when the same vibration signal is transmitted with different vibration impulse durations, (**a**) Vibration impulse duration = 0.1 s; (**b**) vibration impulse duration = 0.2 s.

To minimize the effect of the echoes of the medium where the smartphone is placed, we generate an impulse-like vibration instead of continuous vibrations. Empirically, we set the vibration time for 0.2 s in a single frame. This is the smallest time that can guarantee a reasonable result. If the vibration time is shorter than 0.2 s, the variation of vibration gets smaller, and it becomes hard to decode. As shown in Figure 6, when the vibration time is 0.1 s, not only the amplitude of a raw acceleration reading gets reduced, but the peak exists at only one side, reducing the standard deviation of a frame. We arrived at a similar result when the sampling frequency was higher. As a result, a bit 1 often gets incorrectly decoded as 0. In conclusion, an impulse of a 0.2 s-long vibration is the optimal value in terms of energy efficiency and minimizes the effect of echoes for the current Android platforms.

### 4.6. Adaptive Threshold Adjustment

As noted in Section 3.3, the performance of *VibeComm* depends on the choice of the threshold value used in the signal detection. We have tested three different adaptive threshold adjustment methods for *VibeComm*, and they are: (1) surface detection; (2) thresholding using the range of standard deviations; and (3) the robust likelihood ratio test. The default option of a fixed threshold is tested against the adaptive threshold adjustment methods in Section 6.

#### 4.6.1. Surface Detection

**Figure 7.**
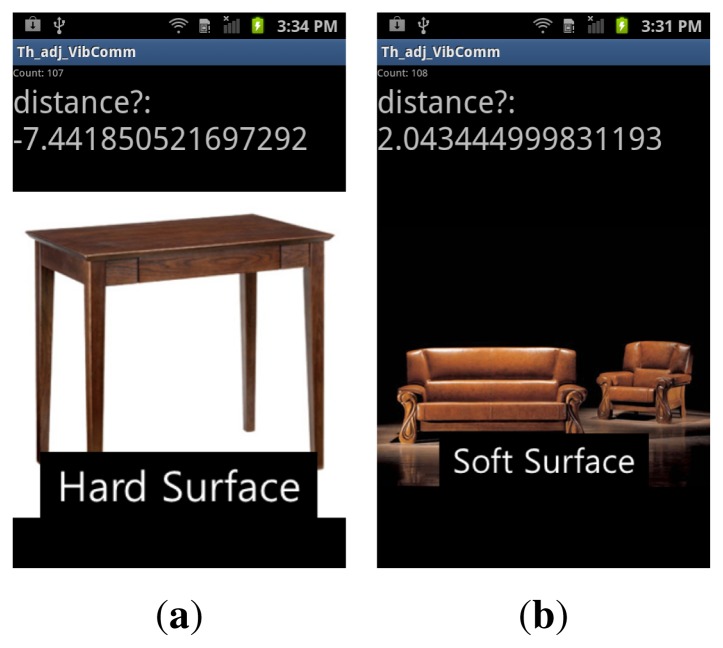
Surface detection using the method proposed in [18]. The resulting SVM scores are displayed, (**a**) Hard surface detection; (**b**) soft surface detection.

In [18], Cho *et al.*, proposed an efficient method to detect the contacting surface type using smartphones by generating vibrations and reading responses from the surface using accelerometers. Their method classified the surface into six different types using a small number of features constructed from accelerometer readings. We applied the method proposed in [18] to detect the rigidness of the communication medium. We partitioned the training set into two groups: Hard surface and soft surface. A two-class support vector machine (SVM) [19] is trained to classify whether the surface is hard or soft. An example is shown in Figure 7. Usually, the SVM declares the test sample in one class if the score computed by the SVM is larger than or equal to zero and the other class if the score is less then zero. However, we used the trained two-class SVM to label the surface as three types using the score computed by the SVM: Hard, medium and soft. If the score is less than −3, the communication medium is categorized as a hard surface. If the score is between −3 and 2, we label the communication medium as a medium-stiff surface. For the score larger than two, the communication medium is classified as a soft surface. These values are selected empirically from a number of trials on different objects, and we have obtained the following thresholds: α_hard_ = 0.01, α_medium_ = 0.008, and α_soft_ = 0.006. For a soft surface, a lower threshold is required, since the vibration dissipates easily on softer objects. However, a lower threshold is susceptible to external disturbances. For a hard surface, a higher threshold is used to improve the robustness against external disturbances.

#### 4.6.2. Thresholding Using the Range of Standard Deviations

While the adaptive threshold adjustment using surface detection changes its threshold value based on the rigidness of the communication medium, the threshold is adjusted at receivers without considering the relationship between the transmitter and receivers, such as the communication distance. In order to consider the relationship between a transmitter and receivers, we can adjust the threshold by measuring the range of standard deviations of normalized accelerometer readings from a one frame-sized pilot signal. Receiving devices compute the standard deviation σ_1_ of the transmitted vibrations. Based on the standard deviation *σ*_0_ of accelerometer readings, when there is no incoming vibration signal, we can find out the range of the standard deviation of the vibration signal, and we set the threshold to the middle point of the range. This approach takes into consideration the distance between the transmitter and the receivers and external disturbances. The resulting threshold value is set as follows:

$$\alpha_{\text{range}}=\frac{1}{2}\left(\frac{\sigma_{1}}{\mu_{1}}+\frac{\sigma_{0}}{\mu_{0}}\right)$$

where *μ*_1_ is the mean of the normalized accelerometer readings when vibrations are received and *μ*_0_ is the mean when there is no vibration signal.

#### 4.6.3. Robust Likelihood Ratio Test

Even though the use of the range of standard deviations can improve the decoding performance, it is not robust against outliers, which may be introduced when the threshold value is determined. In order to reduce the effect of outliers, we propose a robust likelihood ratio test for *VibeComm*. Before communication starts, the transmitter transmits a known pilot signal of seven superframes, which contains 49 active frames. We assume that the vibration signal has the Gaussian distribution. When a bit 1 is sent, the received signal is assumed to be distributed from the Gaussian distribution with mean *μ*_1_ and variance $\sigma_{1}^{2}$. When a bit 0 is sent, the received signal is assumed to be distributed from the Gaussian distribution with mean *μ*_0_ and variance $\sigma_{0}^{2}$. Since the receiver knows the pilot signal, we can estimate all parameters, *i.e.*, *μ*_0_, $\sigma_{0}^{2}$, *μ*_1_ and $\sigma_{1}^{2}$, from the received signals. From our estimates, we can find the threshold that makes the likelihood ratio 0.5. However, a simple likelihood ratio test is susceptible to outliers. Hence, we adapted the random sample consensus (RANSAC) method [20] to robustly estimate signal distribution parameters.

## 5. Implementation

**Figure 8.**
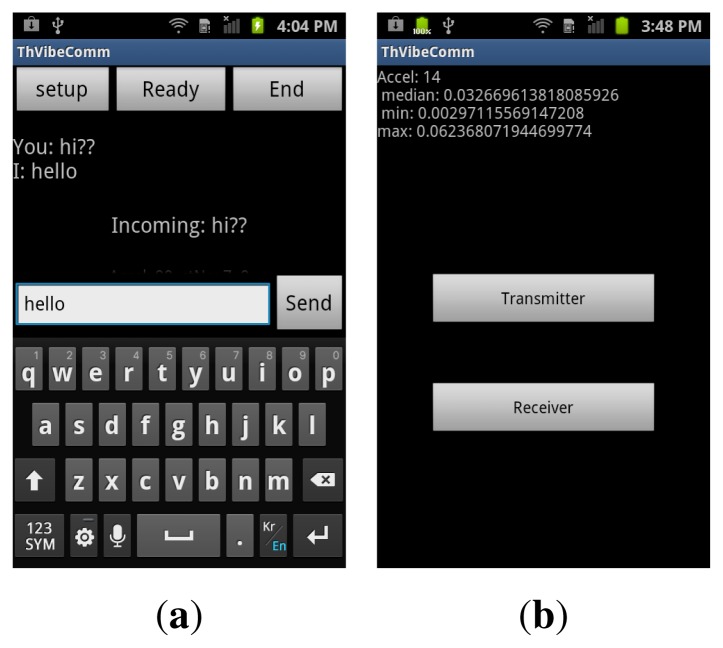
Screen shots of the *VibeComm* prototype. (**a**) Sending and receiving; (**b**) threshold adjustment.

Based on the method described in previous sections, we have developed a *VibeComm* prototype as an Android application. Figure 8 shows screen shots of the developed Android application prototype. The application has three buttons: *Setup*, *Ready* and *End*. When the application acts as a transmitter, it will proceed as follows. It takes an input string from a user and converts it into a series of characters (see Figure 8a). Each character is converted into a superframe whose active frame field contains a seven-bit ASCII code for the character. Finally, the transmitting smartphone vibrates according to the content of the constructed superframe. On the other hand, when the application receives a signal, it starts sampling accelerometer readings when the *Ready* button is pressed (see Figure 8a). The receiver starts detecting movements of the medium using the method described in Section 4.3 and decodes incoming messages. The decoded packets are converted into ASCII codes, and the string is displayed on the screen. The threshold is adjusted before the transmission of data. When no threshold adjustment method is selected, it uses a default threshold value (*α* = 0.005). The user can choose a different adaptive threshold adjustment method by pressing the *Setup* button. Figure 8b shows a screen shot of one of threshold adjustment methods while the application is being configured.

As mentioned earlier, the sampling rate of an accelerometer in an Android platform can be set to one of the following four options: *SENSOR_DELAY_NORMAL* (6± 1 Hz), *SENSOR_DELAY_UI* (17± 1 Hz), *SENSOR_DELAY_GAME* (50 ± 1 Hz) and *SENSOR_DELAY_FASTEST* (100 ± 1 Hz). The stated frequencies are measured empirically. Since the vibration transmission rate is low, a low sampling rate is sufficient for our current implementation, and it also reduces the power consumption. However, the sampling rate of 6 ± 1 Hz using *SENSOR_DELAY_NORMAL* turned out to be too low, and we have noticed that vibration signals are often lost. Hence, the next lowest sampling rate of 17 ± 1 Hz using *SENSOR_DELAY_UI* is used in our prototype.

## 6. Experimental Results

We have tested *VibeComm* under various situations by changing communication medium objects, the detection threshold value, the distances between a transmitter and a receiver, ambient noise levels and different communication directions. We have also demonstrated that an adaptive threshold adjustment method can be used to improve the performance of *VibeComm*. Unless noted otherwise, the detection threshold is set to 0.005.

### 6.1. Communication Medium Objects

**Figure 9.**
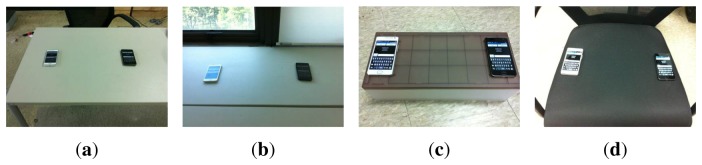
Different communication medium objects used in experiments. (**a**) wooden table; (**b**) metal shelf; (**c**) plastic shelf; (**d**) cushioned chair.

We first tested *VibeComm* using diverse communication medium objects, including a wooden table, a metal shelf, a plastic shelf and a cushioned chair, as shown in Figure 9.

For all experiments, a test string of 100 characters is transmitted three times; hence, a total of 300 characters are transmitted, and the performance is measured based on the decoded bitstream and characters. Note that one character corresponds to one packet in our current implementation, and the terms character or packet are used interchangeably in this paper. For this experiment, the distance between a transmitter and a receiver is 50 cm.

The packet delivery rate and bitwise accuracy are shown in Table 1. The packet delivery rate is computed as the ratio between the number of correctly decoded packets and the total number of transmitted packets. The bitwise accuracy is computed as the percentage of correctly decoded frames. Without an error correction code, the packet delivery rate is always smaller than the bitwise accuracy. Since the threshold value *α* = 0.005 is relatively ideal for hard surfaces, the performance on a metal shelf and a cushioned chair was not as good as the performance on a wooden table. However, the bitwise error shows relatively high performance, and this means that the performance can be improved when an error correction code is used to fix corrupted bits in a packet, e.g., a simple error-correction coding scheme, such as the Hamming(7, 4) code [21], can be used. However, we have not explored this option in our current implementation, since the introduction of an error correction code will further reduce the transmission rate. Hence, a better approach to fix bitwise errors is to adaptively find a good detection threshold, as discussed below. Furthermore, we can opportunistically transmit signals when the communication medium object is stable and the ambient noise level is low.

### 6.2. Detection Threshold

The detection threshold *α* plays an important role in *VibeComm*, as the communication environment can be highly diverse. When the threshold is low, it is easier to detect a vibration signal on the surface, but it is more sensitive to disturbances. On the other hand, when the threshold is high, it is more robust against disturbances, but it may miss actual transmitted signals. Hence, finding the right value for the communication medium object and the surrounding disturbance level is the key to the success of *VibeComm*.

**Figure 10.**
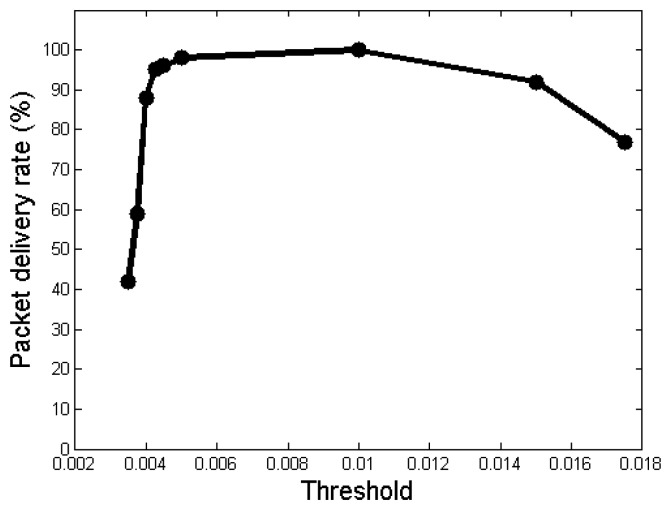
The packet delivery rate as a function of the threshold *α* on a wooden table. The distance between a transmitter and a receiver was 50 cm.

The experimental results with respect to varying the threshold are shown in Figure 10. The experiment was conducted on a long narrow wooden table, and the distance between a transmitter and a receiver was set to 50 cm. As shown in Figure 10, the highest accuracy is achieved when *α* = 0.01 with an accuracy of 100%. However, this does not mean that 0.01 is the best threshold value for all cases or when the communication medium is a wooden table. In fact, we have found that the detection threshold value between 0.005 and 0.015 is good for objects that can be classified as a hard surface. In this experiment, when the threshold is lower than 0.0035, the receiver becomes too sensitive and detects movements when the transmitter has not transmitted a vibration signal. On the other hand, when the threshold is larger than 0.0175, the receiving device becomes too insensitive and even misses beacon frames. The packet delivery rate increases up to *α* = 0.01 and decreases afterwards. This shows that there is an optimal value for *α*, and an adaptive threshold adjustment is required, which is the topic of Section 6.6.

### 6.3. Communication Distance

**Figure 11.**
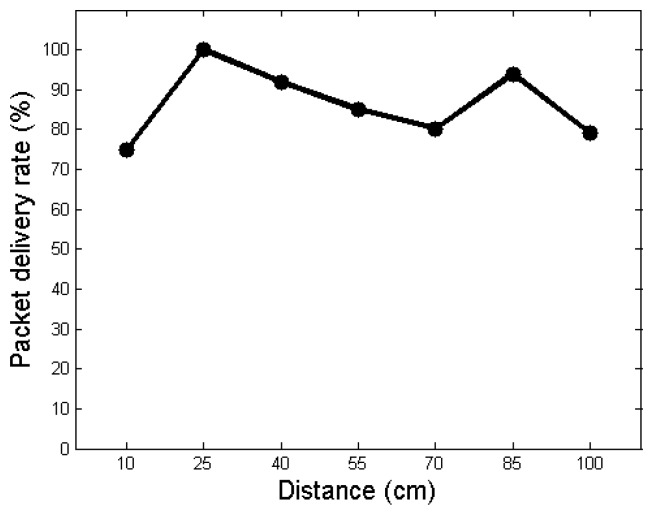
The packet delivery rate as a function of the communication distance between a transmitter and a receiver.

In this section, we tested *VibeComm* by varying the distance between a transmitter and a receiver. The minimum possible distance between two smartphones is about 10 cm, since distances are measured between centers of smartphones. As shown in Figure 11, when the distance between two devices is 25 cm, the packet delivery rate achieves the highest value (100%) and decreases as the distance increases. A surprising finding is that the packet delivery rate is low when the separation between two devices is 10 cm, the shortest distance we have tried in this experiment. One possible reason for this can be the interference from the receiving device. When two devices are placed too close, the receiving device can change the pattern of vibrational motion, as it can act as a barrier against the propagation of vibration and reflected vibrations interfere with transmitted signals. The next interesting fact is that the packet delivery rate is high when the distance is 85 cm. We believe that this is due to the configuration of the table used in the experiment, and reflected vibrations from boundaries might have been positively correlated with the original vibration signal, improving the magnitude of received vibration signals.

### 6.4. Ambient Noise

**Figure 12.**
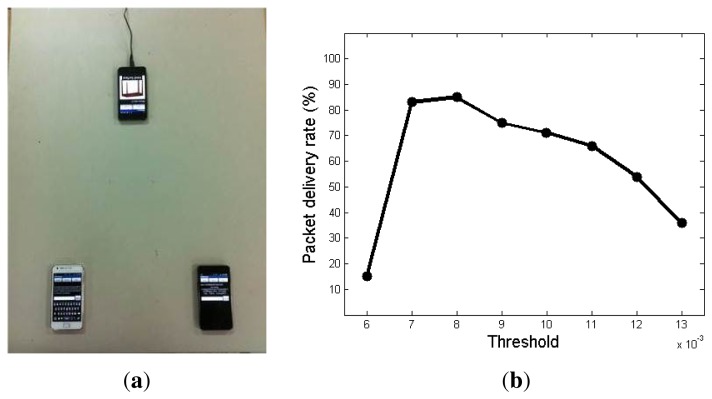
The experimental setup to test the effect of the detection threshold on ambient noise. The transmitter is located on the left bottom, and the receiver is located on the right bottom. The noise source is placed at the top of the photo. The packet delivery rate as a function of the detection threshold under the presence of a noise source. (**a**) Overall setting; (**b**) packet delivery rate under noisy conditions.

Since vibration signals are used for communication in *VibeComm*, any disturbance on the communication medium object can corrupt the transmitted signal. For instance, tapping the communication medium can generate decoding errors, and a single bit error can result in an incorrectly decoded packet without an error correction code. A smartphone is used as a noise source, and it generates repeating vibration patterns (one second of continuous vibration and one second of rest). The distance between the sending device and the receiving device was 30 cm, and the distance from the receiving device to the noise source was 40 cm, as shown in Figure 12a.

Under the setting shown in Figure 12a, we have evaluated the effect of the detection threshold against ambient noise. The packet delivery rates are computed for different detection threshold values. When the detection threshold value is larger than the noise variation and less than the variation of vibration signals generated by the transmitter, we can get the result with reasonably high accuracy. Figure 12b shows that when the detection threshold is lower than 0.006, the accuracy is very low to be used in practice. On the other hand, as the detection threshold gets larger, the packet delivery rate gets lower, showing a similar trend as the experiment with varying communication distances.

### 6.5. Communication Direction

**Figure 13.**
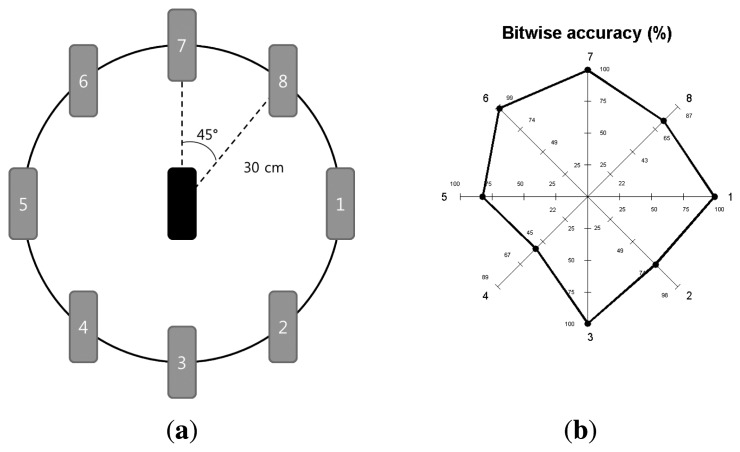
The black rounded-edge rectangle in the middle is the transmitter, and tested locations of the received are shown as gray rounded-edge rectangles. Locations are labeled to make the reference easier. Bitwise accuracy at different communication directions from the transmitter. The locations of the receiver are shown in Figure 13a. (**a**) Overall setting; (**b**) bitwise accuracy.

Since there is a number of components in a smartphone and the placement of a vibrator and an accelerometer can influence the performance of *VibeComm*, we have tested the effect of the communication direction between a transmitter and a receiver. As shown in Figure 13a, we have tested eight different directions by varying the placement of the receiver with respect to the transmitter. The resulting packet delivery rate is shown in a star diagram in Figure 13b. An interesting fact is that the packet delivery rate varies depending on the communication direction. We can achieve high accuracy at Locations 1, 3, 6, and 7. However, the packet delivery rates were low in other locations, while the experimental condition was the same, except the communication direction. This shows that the proposed vibration-based communication method is easier when the receiver is on the right-hand side. We believe that this is due to the placement of a vibrator and an accelerometer inside the smartphone used in the experiment, and the result may vary if a different model is used.

### 6.6. Adaptive Threshold Adjustment

**Figure 14.**
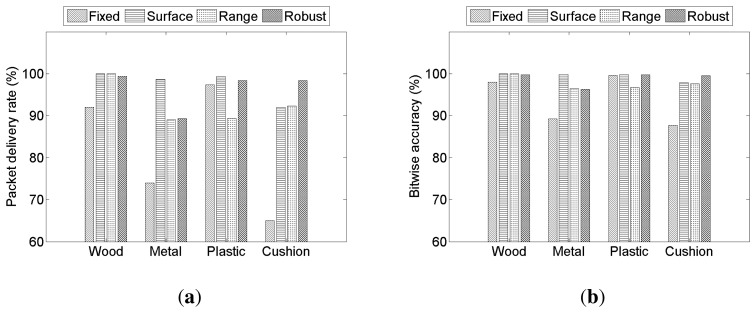
Comparison of different thresholding methods from Section 4.6: *Fixed* (a fixed threshold of *α* = 0.005); *Surface* (thresholding using surface detection); *Range* (thresholding using the range of standard deviations); and *Robust* (robust likelihood ratio test). (**a**) Packet delivery rate; (**b**) bitwise accuracy.

We have compared three different adaptive threshold adjustment methods described in Section 4.6, and they are denoted by *Surface* (thresholding using surface detection from [18]), *Range* (thresholding using the range of standard deviations) and *Robust* (robust likelihood ratio test). The proposed thresholding methods are compared to the case with a fixed threshold, which is denoted by *Fixed* (a fixed threshold of *α* = 0.005), on various communication mediums. The experiment results, including both packet delivery rates and bitwise accuracies, are shown in Figure 14.

All three proposed methods show better performance than using a fixed threshold. The *Surface* method shows better packet delivery rates and bitwise accuracies than *Fixed*. In particular, accuracies on a metal shelf and a cushioned chair have improved significantly. For instance, the bitwise accuracy of a cushioned chair was 87.71% using *Fixed*, but with the *Surface* method, the accuracy is improved to 97.91%. However, the surface detection does not consider the relationship between the transmitter and the receiver. The threshold adjustment methods *Range* and *Robust* are based on the received signals; hence, the threshold values obtained by those two methods are based on the relationship between the transmitter and the receiver.

**Figure 15.**
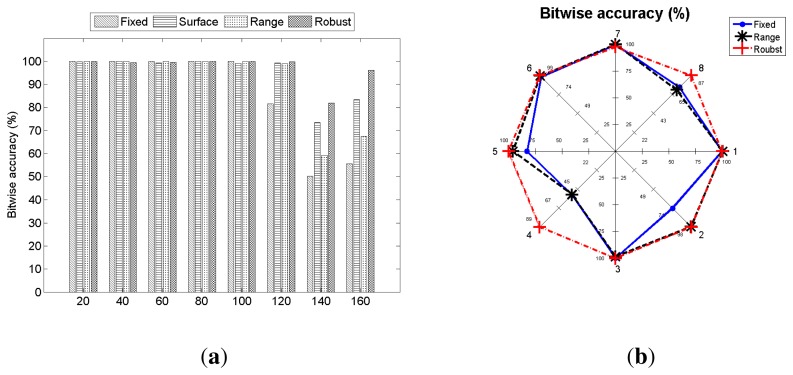
Bitwise accuracies over different distances between the transmitter and the receiver under three different adaptive threshold adjustment methods. Bitwise accuracy at different communication directions from the transmitter when different adaptive threshold adjustment methods are used. The locations of the receiver are shown in Figure 13a. (**a**) Different distance; (**b**) different direction.

The *Range* method shows an improvement from *Fixed*, but its accuracy is less than the *Surface* method. We believe that this is due to the existence of outliers in the pilot signal when the threshold value is determined by the *Range* method. This can be seen from the performance of the *Robust* method, which shows good performance in most cases. It can be seen from Figure 15 that *Surface* performs well when the communication medium is a thin object (metal), and *Robust* works well for a soft object (cushion). Since the *Surface* method is not adjusted for varying communication distances, we believe a more reliable communication can be possible on many different types of objects and conditions if the *Surface* method is integrated into the *Robust* method.

We have also compared bitwise accuracies under three different threshold adjustment methods to *Fixed* at different distances between a transmitter and a receiver. The result is shown in Figure 15a, and it is easy to verify that *Robust* outperforms *Fixed*, *Surface* and *Range*. The *Robust* thresholding method maintains 100% accuracy up to 120 cm and achieves higher rates at 140 cm and 160 cm, compared to three other methods.

We reexamined the effect of the communication direction under different adaptive threshold adjustment method, and the result is summarized in Figure 15b. The plot shows results from three different detection thresholding schemes: *Fixed*, *Range* and *Robust*. The threshold adjustment method using surface detection from Section 4.6.1. is not used, since the communication medium is the same. As shown in Section 6.5, the packet delivery rate varies depending on the communication direction. The effect of the communication direction is significantly reduced by using the *Robust* method, showing its robustness.

## 7. *VibeComm* Demonstration

**Figure 16.**
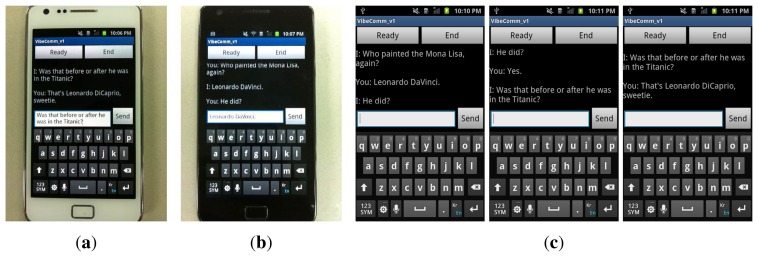
A demonstration of the two-way communication for exchanging text messages. *I* denotes a message sent from the device, and *You* denotes a received message. (**a**) Device 1; (**b**) Device 2; (**c**) a conversation displayed on Device 1.

We first demonstrated *VibeComm* for two-way communication between two smartphones with a 50-cm separation. A short conversation between two smartphones is demonstrated, and screen shots from the demonstration are shown in Figure 16c. We were able to successfully exchange text messages between two devices.

**Figure 17.**
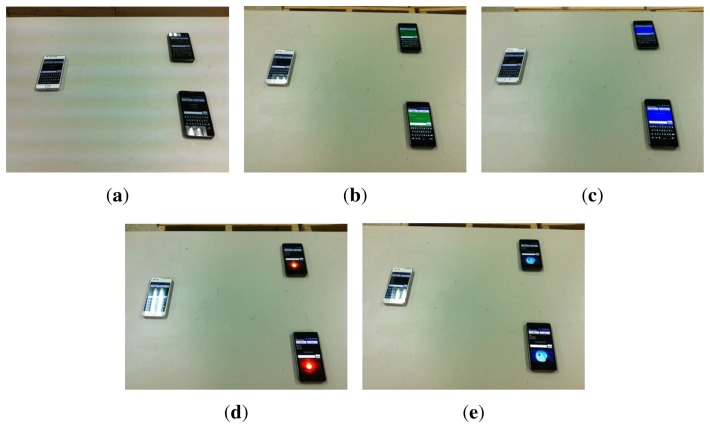
One-to-many communication using *VibeComm*. Upon receiving a command from the transmitter on the left, the receiving devices change their background simultaneously from the blank initial background to the background shown above. (**a**) Initial background; (**b**) green background; (**c**) blue background; (**d**) Sun; (**e**) Moon.

Next, we examined the one-to-many communication capability of *VibeComm*. Instead of transmitting text messages, we have implemented a simple application using *VibeComm* to change the background images of receiving phones simultaneously from a command sent from a transmitting phone. A total of four different colors and ten different pictures can appear at receivers based on the transmitted signal. A vibrational signal from a single transmitter changes the backgrounds of two receiving devices in this demonstration. This example illustrates that *VibeComm* can be used to configuring a number of devices simultaneously without using radios, as discussed earlier. Figure 17 shows some photos taken from the demonstration.

## 8. Discussions

The major limitation of *VibeComm* is its long communication time, which is about 0.09 packets per second. However, the transmission speed can be improved if a vibrating device with higher frequency is embedded into smartphones. We believe that once vibration is adapted as another way to communicate using smartphones, the smartphone manufacturers will equip their smartphones with more sophisticated vibrators and accelerometers with precise sampling frequencies. If faster transmission is possible, we can also use an error correction code to improve the packet transmission rate.

A related issue is the variations in vibrators and accelerometers of the same model. When we compare two smartphones of the same model, accelerometer readings are different for the same vibration sequences, and the strength of vibration generated by each device was also different. Hence, in order to make *VibeComm* practical, it is necessary to adjust the vibrating strength of sending devices and calibrate the accelerometer of each device. We believe this can be solved more easily as more sophisticated vibrators and accelerometers are incorporated into smartphones.

## 9. Conclusions

This paper introduces a novel communication method without using radios. It utilizes built-in vibrators and accelerometers of smartphones that are currently available in all smartphones. The proposed method can be an alternative communication method when general types of radio communication methods are not available. We have implemented a system that can send and receive text information using vibration. For successful communication, we have applied several adaptive threshold adjusting methods and tested the performance of a *VibeComm* prototype under various conditions, showing the feasibility of *VibeComm*.

## Figures and Tables

**Table 1.** Packet delivery rates and bitwise accuracies on four different communication medium objects.

	**Packet Delivery Rate (%)**	**Bitwise Accuracy (%)**
**A**	92.00	98.00
**B**	74.00	89.24
**C**	97.33	99.57
**D**	65.00	87.71

**(A)** wooden table; **(B)** metal shelf; **(C)** plastic shelf; **(D)** cushioned chair.
